# The phenolic and flavonoid content and biological activity of Curcuma (*Curcuma xanthorrhiza*) fractions with different solvent polarities

**DOI:** 10.5455/javar.2025.l886

**Published:** 2025-03-25

**Authors:** Ucop Haroen, Syafwan Syafwan, Kiki Kurniawan, Agus Budiansyah, Nilawati Widjaja, Saitul Fakhri

**Affiliations:** 1 Faculty of Animal Husbandry, Jambi University, Jambi, Indonesia; 2 Reseach Center for Vaccine and Drugs Development, National Research and Innovation Agency, Bogor, Indonesia; 3 Animal Husbandry Study Program, Faculty of Agriculture, Insan Cendekia Mandiri University, Bandung, Indonesia

**Keywords:** *Curcuma xanthorrhiza*, antioxidant, antibacterial, MIC, MBC

## Abstract

**Objective:**

This study aimed to identify the impact of variations in solvent polarity on the solubility of secondary metabolite compounds, which were correlated as antioxidant and antibacterial agents for *Escherichia coli* (ATCC 11725), *Salmonella sp.* (ATCC 22504), *Staphylococcus aureus* (ATCC 11526), and *Bacillus subtilis* (ATCC 11626).

**Materials and Methods:**

A total of 500 gm of Temulawak flour (*Curcuma xanthorrhiza*) was macerated gradually using the step gradient polarity (SGP) technique, where the solvents (methanol, ethyl acetate, and n-hexane) used had different levels of polarity. The extracting process of secondary metabolite components of Temulawak flour began with the use of non-polar solvents, semipolar solvents, and polar solvents, respectively. Each soaking process was completed for 5 × 24 h.

**Results:**

From the Temulawak extract with different levels of solvent polarity, 76.048 gm were obtained for the methanol fraction, 106.242 gm for the ethyl acetate fraction, and 154.575 gm for the n-hexane fraction. The results of the antioxidant activity test showed that the ethyl acetate fraction had the highest inhibitory value for antioxidant activity of 50% (IC50) 22.59 mg/l. The results of this research’s *β*-carotene content were 0.0865 gm. Ethyl acetate fraction was known to have good activity from the antibacterial activity test. The inhibition zone of *E. coli* and *B. subtilis* bacteria was 11.5 ± 0.71 with a minimum concentration of 3.13 mg/ml. The inhibition zone of *S. aureus* was 10.5 ± 0.17 with a minimum inhibitory concentration (MIC) of 6.25 mg/ml, while the inhibition zone for *Salmonella* sp. was 8.125 ± 0.35 with a MIC of 37.50 mg/ml.

**Conclusion:**

All fractions have moderate antibacterial activity, yet the ability of the ethyl acetate fraction of Temulawak extract was higher than the methanol and n-hexane fractions.

## Introduction

Temulawak *(Curcuma xanthorrhiza)* is a medicinal plant belonging to the *Zingiberaceae *family originating from Indonesia and spread across Southeast Asia, such as Malaysia, Thailand, the Philippines, China, India, Japan, Korea, and several countries in Europe [1]. In Indonesia, the *Temulawak *plant is used as an ingredient to make traditional drinks or herbal medicine. *Temulawak* is produced from rhizomes or tubers and can be consumed as a whole or put into traditional drinks [2]. Several studies reported that parts of Temulawak, such as rhizomes or leaves, have been utilized to cure several diseases and are traditionally used to protect the liver from toxic compounds such as carbon tetrachloride and acetaminophen [3]. Many toxins or chemicals can cause negative effects on the liver called hepatotoxins; therefore, Temulawak can be used as a natural ingredient to help maintain liver health [4].

A considerable number of pharmacological properties of Temulawak have been reported, such as antibacterial, anti-implantation, anti-stress, and anti-cancer [5,6]. Temulawak rhizomes can generally be used as traditional medicine; hence, this research used Temulawak rhizomes as samples to evaluate various types of secondary metabolite compounds that would be used as additional feed ingredients that function as anti-stress agents in livestock.

Oxidative stress in livestock, especially poultry, can be caused by heat stress. Heat stress causes an increase in free radicals. Oxidative stress in poultry elicited by high concentrations of free radicals is the main factor of various diseases. The livestock body has many ways to fight oxidative stress, one of which is by producing antioxidant compounds either produced naturally or obtained from their feed as additional feed ingredients [7] to increase the immune response of the livestock’s body and to reduce the risk of degenerative diseases. Therefore, administering Temulawak extract is expected to improve the health of livestock, especially poultry that undergoes stress. Temulawak rhizomes, which contain secondary metabolite compounds and natural antioxidants, have been widely used and recognized for their benefits [8]. Akinola et al. [9] reported that 3 out of 10 species of plants in the *Zingiberaceae* family have high antioxidant activity. They are methanol extracts of turmeric (*Curcuma longa*), ginger (*Zingiber officinale*), and Temulawak (*Curcuma xanthorrhiza*). Furthermore, Kasai et al. [10] reported that the methanol extract of Temulawak provides higher antioxidant activity compared to the Temulawak extract from phosphate and water solvents. In addition, the report revealed that the antioxidant activity of the methanol extract of Temulawak is higher compared to the methanol extract of *Curcuma aromatica* and *Curcuma zedoaria*.

The examination of antioxidant activity capabilities using 2,2-diphenyl-1-picrylhydrazyl (DPPH) reagent as a free radical scavenger has been carried out by several researchers [6,11]. Diverse methods of extracting secondary metabolite compounds found in herbal plants have been carried out to test the antioxidant activity [12,13]. However, until now, none of these have been based on the chemical properties of the solvent’s polarity variation.

The use of organic solvents in the extraction process of secondary metabolite compounds in Temulawak is grounded in the chemical properties of the solvent. Based on these chemical properties, the isolation process of secondary metabolite compounds is conducted based on differences in the polarity levels of the organic solvents used. Differences in organic solvent polarities allow us to identify the types of secondary metabolites contained in Temulawak so that the types of secondary metabolites based on the level of polarity of the solvent used can be grouped. Besides, the type of biological activity that will be applied as a natural feed additive to livestock can be determined. In this case, using different polarities of organic solvents in this research is intended to isolate the secondary metabolite compounds with antioxidant activity, such as phenolics or flavonoids. Apart from that, antibacterial activity testing was also carried out based on the type of secondary metabolites contained in the Temulawak samples.

## Materials and Methods

### Ethical approval

It was determined that ethical approval would not be necessary as the study did not involve the use of any animals.

### Research tools and materials

A total of 2 kg of fresh Temulawak rhizomes were obtained from Jambi, Jambi province, Sumatera, Indonesia, to be dried in the oven at 40°C for 5 × 24 h. Hereafter, the dried Temulawak rhizomes were ground to obtain 500 gm of Temulawak flour. This research used several types of organic solvents, such as n-hexane, ethyl acetate, and methanol, which were used for the extraction process of natural compounds from the Temulawak flour samples obtained from Bratachem, Ltd. Indonesia. Standard gallic acid and quercetin standards were obtained from Merck, Ltd. Meanwhile, the free radical DPPH was gained from Sigma Aldrich, Ltd., and calcium chloride was received from Merc, Ltd., as well as the Whatman filter paper (Sigma Aldrich, Ltd.). The bacterial strains used in this research, namely *Escherichia coli* (ATCC 11725), *Salmonella sp.* (ATCC 22504), *Staphylococcus aureus* (ATCC 11526), and *Bacillus subtilis* (ATCC 11626), were obtained from the microbiology laboratory of the Bogor Agricultural University.

### Preparation of C. xanthorrhiza rhizome samples

This research employed the gradual maceration method with the step gradien polarity (SGP) technique, where the solvents used have different levels of polarity. This SGP technique is expected to thoroughly extract the secondary metabolite components in the samples of Temulawak flour [5]. The extracting process of secondary metabolite components of *Temulawak *flour began with the use of non-polar solvents (n-hexane), semipolar solvents (ethyl acetate), and polar solvents (methanol), respectively. Each soaking process was completed for 5 × 24 h.

### Maceration process of C. xanthorrhiza rhizome samples

A 600 gm sample of dry powder of Temulawak rhizome (*Curcuma xanthorrhiza*) was macerated using the SGP method, which was initiated by non-polar to polar solvents gradually so that three fractions with different polarities could be obtained. The weight of each fraction that had been obtained from the maceration process and had been concentrated using a rotary evaporator was calculated so that the weight of each fraction was obtained. The methanol fraction was dark brown, whereas the color of the ethyl acetate fraction was brownish orange, and the n-hexane fraction was orange.

### Phytochemical screening

The determination of the secondary metabolite content within samples of Temulawak rhizomes (*Curcuma xanthorrhiza*) was based on the method explained by Twaij and Hasan [6].

### Antibacterial activity testing

Antibacterial activity testing of a compound can be carried out using several methods, including the diffusion and dilution methods. The disc diffusion method is a method commonly used in laboratories for clinical antibacterial susceptibility testing. The antibacterial activity test was accomplished by measuring the diameter of the inhibition zone. This method has some advantages, such as being cheap, straightforward, and easy to interpret the results [14]. Meanwhile, the dilution method can be used to determine the minimum inhibitory concentration (MIC) and the minimum bactericidal concentration (MBC). The minimum concentration was determined by the microdilution dilution method, which was carried out by double dilution of antibiotics distributed into the appropriate microtiter plate, whereas the MIC was completed visually by looking at the turbidity on the microtiter plate [15]. MBC is indicated by the absence of bacteria growth in the scratches on the cup, designating that each test bacterium died due to the concentration of the test solution [16].

### Antibacterial activity testing with the dilution method

The test began with preparing sterile filter paper with a diameter of 5 mm, and each was impregnated with 10 μl of samples of n-hexane fraction extract, ethyl acetate, and 5,000 ppm methanol, as well as a positive control of 200 ppm tetracycline and a negative control by using dimethyl sulfoxide. Henceforth, the filter paper was placed on an agar surface that had been inoculated with the test bacteria and then incubated at 37°C for 18–24 h. The antibacterial activity was thus evaluated by measuring the diameter of the inhibition zone of test bacteria growth around the paper disc [17].

### MIC test

A sterile microplate consisting of 96 wells, 8 vertical wells AH, and 1-12 horizontal wells 1-12 was prepared and injected with Muller Hinton Broth as much as 100 μl of sample; the tetracycline positive control and negative control were each injected into well A. Next, dilution was carried out from Well A to Well H. The test bacteria were also prepared and diluted in 0.85% physiological saline and adjusted to the 0.5 McFarland standard, or equivalent to ± 1 × 10^8^ CFU/ml. Henceforth, a total of 100 μl of the diluted bacteria were injected into each well. The microplate was hereafter incubated at 37°C for 18–24 h. The MIC was optically observed by determining the smallest concentration that formed a clear zone [15].

### MBC test

The lowest sample concentration that appeared to inhibit bacterial growth was then plated on the MHA medium, and a total of 5 μl was taken to be injected into a petri dish containing the MHA medium. It was then incubated at 37°C for 18–24 h. The MBC is determined based on the lowest concentration that does not show any bacterial growth on the newly inoculated agar plate [16].

### Determination of total phenolic content

The concentration of phenolic compound groups in the Temulawak extract was determined quantitatively by using spectroscopy methods [18]. A total of 1 mg of the extract solution was mixed with 1 ml of a 10% solution of Folin–Ciocalteu reagent, which was then diluted to a volume of 13 ml of distilled water. Next, a total of 5 ml of 7% Na_2_CO_3_ solution was added to the test solution. The mixture was homogenized and stored in a dark room for 2 h. Moreover, 0.5 ml of methanol and 2.5 ml of 10% Folin–Ciocalteu reagent were dissolved in water with 2.5 ml of 7.5% Na_2_CO_3_ prepared as a blank solution and were then stored at 45°C for 45 min. The measurement of absorbance was carried out using a spectrophotometer at λ_max_ = 765 nm. A standard gallic acid solution was prepared using the same procedure, and a calibration curve was created. The concentration of total phenolic was obtained based on the absorbance value gauged on a spectrophotometer. The total phenolic concentration contained in the test extract is expressed in terms of gallic acid equivalent (mg GA/extract).

### Determination of total flavonoid content

Similar to phenolic, spectroscopy methods were employed to calculate the flavonoid levels contained in Temulawak extract [19]. 1 mg of test extract was mixed in 1 ml methanol, and 1 ml of a 2% AlCl_₃_ solution in methanol was then incubated at room temperature for 1 h. Absorbance was determined using a spectrophotometer at λ_max_ = 415 nm. Once the sample incubation process was completed, it was then analyzed to obtain the absorbance value. The calibration curve of the standard solution of quercetin was accomplished using the same procedure. The flavonoid concentration value was determined as mg/ml on the calibration curve. The flavonoid concentration in the test extract was asserted to be equivalent to the standard solution of quercetin (mg Qu/gm extract).

### Determination of β-carotene

A total of 50 gm of Temulawak flour was macerated with a solvent mixture of n-hexane-acetone-calcium salt (CaCl_₂_) in a ratio of 1:1:1. The extraction process of *β*-carotene in the solution was hereupon centrifuged at a speed of 3,000–5,000 rpm for 15 min. The precipitate formed was separated by filtrate; meanwhile, the precipitate containing *β*-carotene compounds was washed with a saturated CaCl_2_ salt solution and separated also by filtrate. The precipitate obtained from the washing process was then dried using a rotary evaporator at a temperature of 40°C [5].

### Evaluation of antioxidant activity

The antioxidant activity of Temulawak extract in binding free radicals (DPPH) was determined quantitatively using the spectrophotometric method by dissolving 1,000 ppm of the stock solution in 1 ml of methanol solvent. Then multilevel dilutions were carried out to obtain concentrations of 10, 50, 100, and 200 ppm. Then, the DPPH solution in methanol with a concentration of 1,000 ppm was added to the test solution whose concentration had been determined. The test solution mixed with DPPH in methanol was stored for 30 min at room temperature (25°C) to reach an optimum reaction condition. The absorbance was determined by using a spectrophotometer at a wavelength of 517 nm. The quercetin solutions whose concentrations had been varied with final concentrations of 5, 15, 25, and 50 ppm were prepared as control solutions. The percentage of inhibition is determined using the equation below, where the IC50 value is determined from the graph of percentage inhibition versus sample concentration using a non-linear algorithm calculation [18].

(%) Inhibition = [1-(As/A0)×100]………… (1)

### Preparation methods of quercetin

Quercetin solution was used as a positive control to determine the total antioxidant activity of Temulawak extract. The preparation of a standard solution of quercetin follows the following protocols. Initially, 10 mg of quercetin crystals were carefully weighed and then put into a 100 ml volumetric flask and completely dissolved in 5 ml of ethanol. Then, the volume of the solution was filled with ethanol to the limit mark or by adding 95 ml of ethanol. The 100 ppm quercetin mother liquor was hereafter differed in concentration by multilevel dilution using a volumetric flask to obtain final concentrations of 5, 15, 25, and 50 ppm.

## Results and Discussions

### Extraction results by maceration

From the extraction process of the Temulawak rhizome with different levels of solvent polarity, it was found that the weights of methanol, ethyl acetate, and n-hexane extracts were 76.048, 106.242, and 154.575 gm, respectively. The use of the SGP method allows optimal extraction of components, and the process is faster without causing problems with the formation of emulsions during separation. The difference in extraction results is influenced by the solubility of secondary metabolites in every solvent used, which is in line with the principle “like dissolves like,” where organic compounds will dissolve in solvents that have similar properties. From this study, it is known that in Temulawak rhizomes (*Curcuma xanthorrhiza*), many secondary metabolite components are semi-polar compared to those that are polar; this is evidenced by the large number of ethyl acetate fraction extracts that are semi-polar. Meanwhile, for non-polar fractions, secondary metabolite compounds that are non-polar will also be dissolved.

### Phytochemical profile of Temulawak rhizomes (Curcuma xanthorrhiza)

The analysis of the phytochemical profile of Temulawak rhizomes was completed for each fraction attained from the maceration process. The three fractions obtained were n-hexane, ethyl acetate, and methanol, and the results of the phytochemical analysis can be seen in Table 1 below. The information regarding the types of secondary metabolites in each fraction can be used as a basis for future research of this study. This research also examined the total phenolic and flavonoid content, as well as the antibacterial activity test on each fraction. The results of phytochemistry that have been carried out are known in the ethyl acetate and methanol fractions containing secondary metabolite compounds that are semi-polar, namely flavonoids and phenolic types. This study focused on semi-polar and polar fractions to determine the total flavonoid and phenolic content contained in each ethyl acetate and methanol fraction.

### Determination of total phenolics

An analysis of total phenolic was done on methanol and ethyl acetate fractions because it had phytochemical reactions on the three extracts of Temulawak (*Curcuma xanthorrhiza*). The analysis used the Folin–Ciocalteu solution by equalizing the concentration of the gallic acid solution. The value of total phenolic was obtained from the measurement results of a spectrophotometer by entering it into the equation of *y* = 0.0527x – 0.2175 *R*² = 0.9994, and it is substituted into the mg GA/extract value. The total phenolic value contained in the methanol fraction and ethyl acetate fraction of the results of this study can be seen in Table 2.

The ethyl acetate fraction has more phenolics (51.8425 ± 0.61 mg GA/extract) compared to the methanol fraction. The total phenolics of the methanol fraction was only 45.2922 ± 0.27 mg GA/extract. The low level of total phenolic contained in the methanol fraction compared to that contained in the ethyl acetate of the Temulawak sample is caused by the semipolar characteristic of the phenolic compound group so that phenolics will dissolve well also in semipolar solvents [5]. First, this result shows that the extraction technique is influential in determining the level of phenolics within a sample of natural elements, particularly for the Temulawak samples. Secondly, it was also found that the group of phenolics is predominantly semipolar; thus, it dissolves well in the ethyl acetate fraction, which is indicated by the higher amount of phenolics in the ethyl acetate than in the methanol fraction [20]. This study provides information that the physical properties of secondary metabolite compounds such as phenolics are determined by the solubility of these compounds in a solvent that has the same polarity properties. In this case, phenolic compounds will dissolve very well in semi-polar solvents to provide high total phenolic values in the ethyl acetate fraction compared to the methanol fraction.

**Table 1.** Phytochemical profile test of methanol, ethyl acetate, and n-hexane.

No	Test parameter	Observation	Methanol fraction	Ethyl acetate fraction	n-hexane fraction
1	Alkaloids	Orange precipitate	Orange precipitate (√)	Orange precipitate (√)	Orange precipitate (×)
2	Phenolics	Dark purple	Purple solution (√)	Purple solution (√)	No purple solution (×)
3	Flavonoids	Red-orange-colored solution	Red-colored solution (√)	Red-orange-colored solution (√)	No red-orange-colored solution (×)
4	Triterpe noids	Red-purple-colored solution	No red-purple-colored solution (×)	Purple-colored solution (√)	Purple-colored solution (√)
5	Steroids	Green-colored solution	No green-colored solution (×)	No green-colored solution (×)	No green-colored solution (√)
6	Saponins	The foam does not disappear after the addition of concentrated HCl	Foam is formed after the addition of concentrated HCl (√)	Foam is formed after the addition of concentrated HCl (√)	Foam is not formed after the addition of concentrated HCl (×)
7	Coumarin	Blue-green fluorescence appears under the 365 nm UV lamp after it is sprayed with 2% NaOH	Blue fluorescence was identified after spraying with 2% NaOH (√)	Yellow-green fluorescence appears after spraying with 2% NaOH (√)	No fluorescence after 2% of NaOH was sprayed (×)

Note: Phytochemical screening was conducted at the Natural Materials Chemistry laboratory of the Chemistry Research Center, National Research and Innovation Agency, 2023.

**Table 2.** The phenolic levels in the ethyl acetate and methanol fractions were expressed as mg GA/extract gallic acid equivalent.

Fractions	Absorbance	Phenolic Levels (mg GA/extract)	Average + SD
Ethyl acetate	1.3546	51.4709	51.8425 ± 0.61
	1.3775	52.2770	
Methanol	1.1883	45.0967	45.2922 ± 0.27
	1.1986	45.4876	

Note: The total phenolics in ethyl acetate and methanol fractions were determined at the Natural Materials Chemistry laboratory of the Chemistry Research Center, National Research and Innovation Agency, 2023.

### Determination of total flavonoids

The analysis of the total flavonoids from the Temulawak samples tested for the positive flavonoid fraction was determined from the results of phytochemical tests with a standard solution of quercetin. In this study, methanol and ethyl acetate fractions were positive for flavonoids. The analysis used a spectrophotometer to calculate the total flavonoid levels of a fraction being tested by substituting the absorbance value obtained from the measurement into the standard curve of Quercetin (*y* = 0.01x + 0.0094 *R*² = 0.9995). Total flavonoid levels of ethyl acetate fraction and methanol fraction of Temulawak (*Curcuma xanthorrhiza*) were expressed in mg Qu/extract. From the analysis of the total flavonoids of the two flavonoid-positive fractions, it was identified that the ethyl acetate fraction contains higher flavonoids compared to the methanol fraction. The total flavonoid of the ethyl acetate fraction was 57.82 ± 0.17 mg Qu/extract, whereas the total flavonoid for the methanol fraction was 47.80 ± 0.37 mg Qu/extract. Flavonoids are secondary metabolite compounds that have semipolar properties; hence, they easily dissolve in semipolar solvents such as ethyl acetate. Meanwhile, the flavonoid group dissolved in polar solvents (methanol) usually contains a glucose group bound as a substitute to the flavonoid framework [21]. The results of the calculation of total flavonoid content contained in both extracts, namely ethyl acetate extract (semi-polar) and methanol extract (polar), obtained information that the ethyl acetate fraction (semi-polar) has a higher value than the methanol fraction (polar). The ability of ethyl acetate extract is directly proportional to the solubility of flavonoid compounds that dissolve in the ethyl acetate fraction (semi-polar) compared to the methanol fraction (polar). This also gives us knowledge that the group of flavonoid compounds dissolved in the ethyl acetate fraction (semi-polar) is a type of flavonoid compound that does not contain sugar groups so that the compound will be semi-polar. A clear description of the total flavonoids of ethyl acetate and methanol fractions from the Temulawak sample can be seen in Table 3.

### Total antioxidant activity

The analysis of antioxidant activity from Temulawak extract concentrated on two fractions that contain polyphenolic compounds, namely the ethyl acetate and the methanol fractions. Compounds containing hydroxy groups, such as phenolic and flavonoid, can stabilize free radicals, which can cause oxidation. In other words, flavonoid and phenolic compound groups can function as natural antioxidants found in Temulawak rhizomes (*Curcuma xanthorrhiza*). Temulawak extract is also known to consist of several compounds that potentially function as free radical scavenging agents, such as curcuminoid, *α*-curcumin, ar-Turmerone, and xanthorrhizol [22]. In this study, DPPH was used as a source of free radicals because DPPH is relatively stable in methanol; thus, it is straightforward to apply in research.

In this study, it was discovered that the activity of the ethyl acetate fraction was better compared to the methanol fraction in capturing free radicals carried by DPPH, which was used as a free radical generating agent. It is evident by the value of IC_50_ developed after testing free radical activity on the ethyl acetate and methanol fractions. Specifically, the ethyl acetate fraction showed a high inhibitory value on antioxidant activity or free radical scavenging of 50% (IC_50_) 22.59 mg/l. This value is powerful enough to inhibit free radical activity in solution. As for the methanol fraction, the inhibitory value was lower at 156.38 mg/l. This high capability of the ethyl acetate fraction in inhibiting free radical activity is greatly correlated with the type of secondary metabolites contained in the fraction and the high amount of phenolic and flavonoid compounds [23]. The presence of both groups of phenolic and flavonoid compounds in the ethyl acetate fraction allows a stabilizing reaction of free radicals brought by DPPH in testing this antioxidant activity. The hydroxyl group (-OH) identified in these two groups can capture free radicals by converting themselves into a stable form with the help of the electron transfer found in the hydroxyl groups found in the framework of phenolic and flavonoid compounds [24]. The antioxidant activity research carried out on both extracts, namely the ethyl acetate extract, which is semi-polar, and polar methanol extract, clear information is provided that the polyphenol compound group is abundant in the ethyl acetate extract (semi-polar) so that it provides an excellent response in inhibiting the development of free radicals produced by radical carrier test compounds, namely DPPH. The ethyl acetate (semi-polar) fraction contained in Temulawak (*Curcuma xanthorrhiza*) rhizome samples is known to contain higher phenolic and flavonoid compound groups than the methanol (polar) fraction.

**Figure 1.**
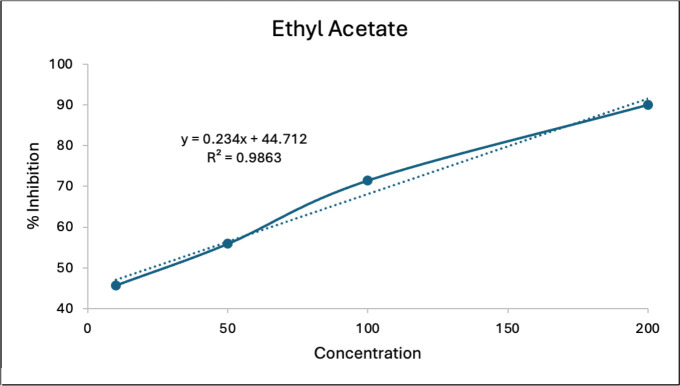
The correlation of inhibition and the concentration of ethyl acetate fraction of Temulawak rhizome.

The value of the antioxidant activity of the ethyl acetate fraction of Temulawak rhizomes is described in the following Table 4. Figure 1 shows that the higher the concentration of ethyl acetate fraction from the rhizome of Temulawak (*Curcuma xanthorrhiza*), the higher the percentage of inhibition.

### Determination of β-carotene

Determining the level of *β*-carotene aims to improve the quality of poultry eggs given that Temulawak flour is used as a feed additive in poultry feed. It is characterized by a change in the color of the poultry egg yolk, which becomes brighter and can increase the concentration of vitamin A in the egg yolk. Isolation and the determination of *β*-carotene level were conducted qualitatively, so information regarding the percentage of *β*-carotene in the samples of Temulawak rhizome was obtained. The higher the level of beta-carotene in poultry feed, the better the quality of eggs produced by the birds. The beta-carotene compound group successfully isolated in this study was a bright yellow–orange solid weighing 0.0865 gm. The isolation process and determining the levels of *β*-carotene from the rhizome of Temulawak or *C. xanthorrhiza* were very much determined by the extraction process, which was adding a mixture of acetone-calcium chloride-n-hexane appropriately so that the *β*-carotene compound that had been extracted dissolved properly in the organic solvent. This process intends to attract all types of *β*-carotene groups to attain maximum results. Furthermore, to obtain maximum extraction results of *β*-carotene precipitate, a centrifugation process was carried out [25].

**Table 3.** The total flavonoid of the ethyl acetate and methanol fractions of Temulawak (*Curcuma xanthorrhiza*).

Fractions	Absorbance	Flavonoids levels (mg Qu/extract)	Average + SD
Ethyl acetate	0.2991	57.94	57.82 ± 0.17
	0.2979	57.70	
Methanol	0.2497	48.08	47.80 ± 0.37
	0.2471	47.54	

Note: The total flavonoids in ethyl acetate and methanol fractions were attained from the Natural Materials Chemistry laboratory of the Chemistry Research Center, National Research and Innovation Agency, 2023.

**Table 4.** Antioxidant activity of ethyl acetate fraction of Temulawak rhizome.

Concentration	Absorbance	% Inhibition	IC_50_
200	0.1985	90.0321	22.5982
100	0.5687	71.4422	
50	0.8780	55.9104	
10	1.0815	45.6914	

Note: Antioxidant activity of ethyl acetate fraction of Temulawak was identified in the Natural Materials Chemistry laboratory of the Chemistry Research Center, National Research and Innovation Agency, 2023

### Antibacterial activity testing with the well dilution method

Antibacterial activity tests in this research were accomplished on all fractions of Temulawak that had been previously obtained during the extraction process. For the three fractions obtained, an antibacterial activity test was completed using four types of bacteria consisting of two groups; they were gram-positive and gram-negative bacteria. The four types of bacteria used in this research were *E. coli* (ATCC 11725), *Salmonella sp.* (ATCC 22504), *S. aureus* (ATCC 11526), and *B. subtilis* (ATCC 11626). The positive control used tetracycline antibiotics with a concentration of 200 ppm, while the negative control used 100% ethanol. This antibacterial research was conducted to see the extent of the inhibition zone using the *well-dilution* method. All fractions of Temulawak gained from the maceration process are tested for their antibacterial activity to calculate the size of the inhibition zone from each fraction on the inhibitory power of growth-tested bacteria [26]. Table 5 provides the results of the antibacterial activity test of Temulawak fractions.

**Table 5.** Antibacterial activity test of Temulawak fractions.

Samples	Diameter of clear zone (mm)			
	*Escherichia coli*	*Salmonella* sp.	*Bacillus subtilis*	*Staphylococcus aureus*
Methanol (MeOH)	10.75	8.00	10.50	9.00
	9.75	8.00	10.75	10.25
	10.625	8.00	10.625	10.50
STDEV	0.17	0.00	0.17	0.88
Ethyl Acetate (EtOAc)	12.00	8.00	12.00	10.25
	11.00	8.25	11.00	10.75
	11.50	8.125	11.50	10.50
STDEV	0.71	0.17	0.71	0.35
n-hexane	10.75	8.00	9.75	9.75
	9.75	7,5	10.75	9.50
	10.25	7.75	10.25	9.625
STDEV	0.71	0.35	0.71	0.17
Control +	16.00	8.375	16.00	14.50
Control -	5.00	5.00	5.00	5.00

Note: Antibacterial activity test was conducted in the Microbiology Laboratory, Research Centre for Vaccine and Drug, National Research and Innovation Agency, 2023.

**Figure 2.**
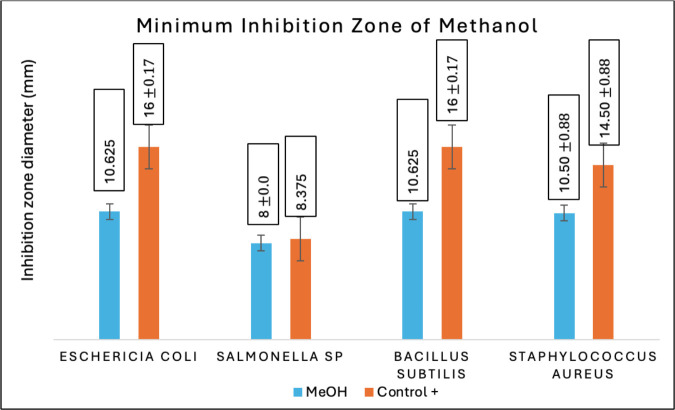
Antibacterial activity test results of the methanol fraction of Temulawak (*Curcuma xanthorrhiza*).

**Figure 3.**
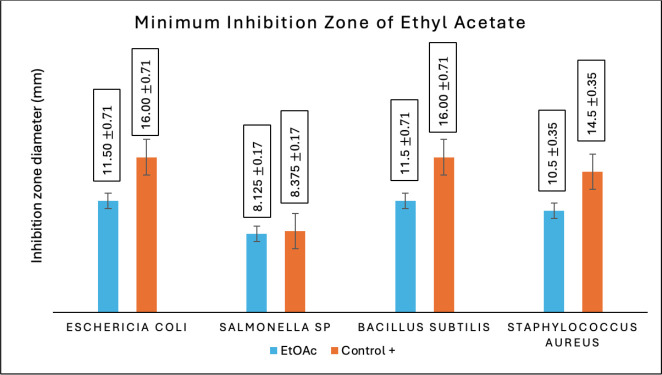
Antibacterial activity test results of ethyl acetate fraction of Temulawak (*Curcuma xanthorrhiza*).

**Figure 4.**
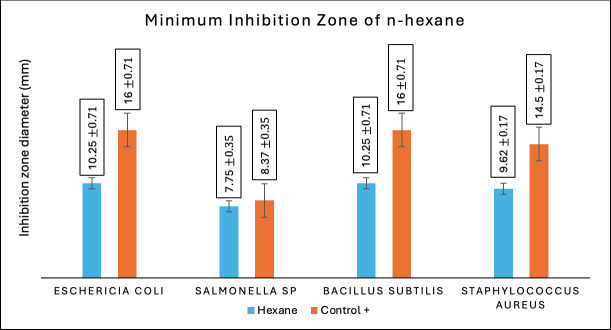
Antibacterial activity test results of the n-hexane fraction of Temulawak (*Curcuma xanthorrhiza*).

Based on the inhibition test of bacterial growth completed on the three fractions of Temulawak, it was found that ethyl acetate has better activity than methanol and n-hexane fractions. The force of ethyl acetate in inhibiting tested bacterial growth is correlated to the type of secondary metabolite consisting in that fraction, such as phenolics and flavonoids [27]. As presented in Tables 2 and 3, the test result indicates that the ethyl acetate fraction contains a higher amount of phenolics and flavonoids compared to the methanol fraction. Therefore, the antimicrobial activity is also high. The Temulawak extract used in this research has a favorable ability to inhibit the growth of test bacteria. The higher the amount of phenolics and flavonoids, the higher the antimicrobial activity [28]. In the present study, it was established that the ethyl acetate fraction (semipolar) exhibited a higher level of antibacterial activity in comparison to the methanol (polar) and n-hexane (non-polar) fractions. Moreover, it was observed that the ethyl acetate fraction (semipolar) resulted in a more pronounced effect on bacterial growth, as evidenced by the wider diameter of the clear zone produced by the disc. The capacity to impede bacterial proliferation is directly proportional to the concentration of secondary metabolite compounds present in the ethyl acetate fraction (semi-polar). Specifically, the phenolic compound group and flavonoids, which are elevated, play a pivotal role in the suppression of test bacteria. The ability of Temulawak extract to inhibit the growth of tested bacteria can be seen in Figures 2–4.

### MIC activity test

MIC activity is defined as the lowest concentration of an antimicrobial agent that impedes the growth of the test microorganism visible on a 96-well microtiter plate. MIC was determined by choosing the first well where no growth was observed after incubation [29]. The examination of the MIC activity test was done to determine the minimum concentration of the three extracts of the Temulawak rhizome sample in inhibiting the growth of the test bacteria. From the MIC examination result, a direct proportional correlation was observed to the inhibition zone test that was carried out using the well diffusion method. The previous test discovered that the ethyl acetate fraction has better activity in inhibiting the growth of test bacteria than the methanol and n-hexane fractions from the Temulawak extract. This was also shown by the results of the MIC test, where the minimum concentration could be used to inhibit the growth of the test bacteria, and this was indicated by the absence of turbidity on the microtiter plate. The ethyl acetate fraction of Temulawak was also found to be the best fraction in the MIC test activity against the growth of gram-positive and gram-negative bacteria. The observation on the microtiter plate was done after 24 h of testing. Meanwhile, the observations on all tested fractions were achieved by looking at the turbidity of the test solution on the microplate.

From the results of the MIC test and the inhibition zone test, *E. coli* and *B. subtilis* were found to be the most susceptible to the ethyl acetate fraction extract with an inhibition zone value of 11.5 ± 0.71 with a minimum concentration of 3.13 mg/ml of the ethyl acetate fraction extract. For *S. aureus *bacteria, the ethyl acetate fraction of Temulawak provides information on the inhibitory zone at 10.5 ± 0.17 with a MIC value of 6.25 mg/ml. However, for *Salmonella sp.*, there was a decline in the inhibitory zone activity of all fractions, where the ethyl acetate fraction had an inhibitory zone value of 8.125 ± 0.35 with a MIC value of 37.50 mg/ml.

From this research, it was found that the ethyl acetate fraction of Temulawak was the best one to be used to inhibit bacterial growth with the minimum concentration used [14]. In determining the minimum inhibitory test value of the concentration of each fraction used it has been established that the ethyl acetate fraction (semi-polar) exhibits higher activity in comparison to the methanol fraction (polar) and the n-hexane fraction (non-polar). The capacity of the ethyl acetate fraction (semi-polar) is closely associated with the content of phenolic compounds and flavonoids present. These compounds have been shown to possess antibacterial properties in this study. The summary of MIC activity values for each Temulawak fraction can be seen in Table 6. The strength of each test fraction in inhibiting the growth of test bacteria can be ranked as follows: ethyl acetate fraction > methanol fraction > n-hexane fraction.

### MBC activity test

The goal of the MBC test of the Temulawak fraction is to observe the ability of each obtained fraction to kill the test bacteria. From this study, it was found that not only could the extract of the Temulawak fraction inhibit the growth of test bacteria, but it could also kill the test bacteria. This is indicated by the concentration value required by the ethyl acetate fraction (semi-polar), which is comparatively low in terms of its bactericidal effect on the four test bacteria in this study. When compared to the methanol fraction (polar) and the n-hexane fraction (non-polar), the ethyl acetate fraction (semi-polar) is shown to be significantly more efficacious. The secondary metabolite compounds contained in the ethyl acetate fraction, such as phenolics and flavonoids, have been demonstrated to possess excellent antibacterial activity.

The ability of Temulawak extract to kill test bacteria can be seen in Table 7. Specifically, the ethyl acetate fraction used in this research had better killing power than methanol and n-hexane fractions. The ability of each fraction to kill the test bacteria was observed after 24 h of incubation. The ability was determined from the lowest concentration attained after carrying out the test. The MBC was analyzed and decided by looking at the level of turbidity of the solution produced.

Table 7 shows that the ethyl acetate fraction is the most active in killing the test bacteria, as seen from the MBC value produced at 6.25 mg/ml for *E. coli* and *B. subtilis* bacteria. For the *S. aureus *bacteria, the MBC value obtained was 12.50 mg/ml, and the MBC value of *Salmonella sp. *was 50.00 mg/ml. The MBC values received in this research designate that the ethyl acetate fraction has good antibacterial activity compared to the methanol and n-hexane fractions. The ability of the ethyl acetate fraction to kill the test bacteria is strongly correlated to the type of secondary metabolite contained in the fraction [30].

This study is still focused on the discovery of fractions with antioxidant and antibacterial activities. This observation is known from the content of secondary metabolites (phenolic and flavonoid compound groups), which act as inhibitors of DPPH free radical activity, added to the test reaction. In addition, the ethyl acetate fraction of Temulawak rhizome (*Curcuma xanthorrhiza*) has antibacterial activity characterized by the ability to inhibit and kill the growth of test bacteria at very low concentrations. To find out more about the types of secondary metabolite compounds that have antioxidant and antibacterial activities, further work is needed in the form of isolation and characterization of pure compounds from active fractions and re-testing of biological activity to prove the ability of the isolated compounds.

**Table 6.** The comparison of MIC activity values of each fraction against the four test bacteria.

MIC (mg/ml)				
	*Escherichia coli*	*Salmonella* sp.	*Bacillus subtilis*	*Staphylococcus aureus*
MeOH fraction	18.75	59.56	37.50	25.00
EtOAc fraction	3.13	37.50	3.13	6.25
n-hexane fraction	62.50	78.12	87.50	75.00
Control +	0.39	0.39	0.39	0.39
Control -	100.00	100.00	100.00	100.00
Documentation	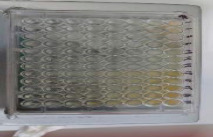	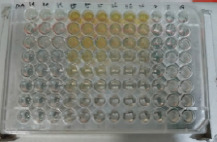	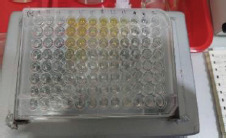	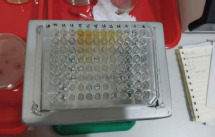
	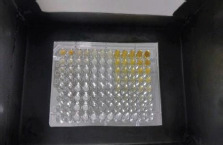	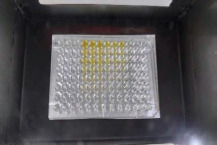	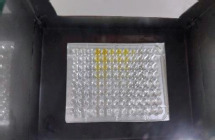	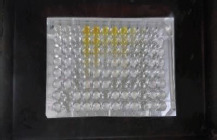

Note: The MIC activity test was conducted in the Microbiology Laboratory, Research Centre for Vaccine and Drug, National Research and Innovation Agency, 2023.

**Table 7.** The value of MBC activity of the three extracts of Temulawak fractions against the test bacteria.

MBC (mg/ml)				
Sample	Bacteria/Concentration			
	*Escherichia coli*	*Salmonella* sp.	*Bacillus subtilis*	*Staphylococcus aureus*
MeOH fraction	31.63	87.50	37.50	25.00
EtOAc fraction	6.25	50.00	6.25	12.50
n-hexane fraction	78.50	112.50	87.50	75.00
Tetracycline	0.625	0.625	0.625	0.625
Methanol	100.00	100.00	100.00	100.00

Note: The MBC activity test was conducted in the Microbiology Laboratory, Research Centre for Vaccine and Drug, National Research and Innovation Agency, 2023.

## Conclusion

Based on the research conducted on Temulawak (*Curcuma xanthorrhiza*) extract obtained using extraction methods with different polarity levels of organic solvents, Temulawak extract is found to contain secondary metabolites that play an important role in biological activities, especially antioxidant activity and antibacterial activity. In this research, Temulawak is found to consist of *β*-carotene in the form of a bright yellow–orange colored solid weighing 0.0865 gm, which is extracted using a solvent mixture of n-hexane-acetone-calcium salt (1:1:1). Moreover, antioxidant activity is affected by the total phenolic and total flavonoid in the ethyl acetate and methanol fractions. Ethyl acetate is the most powerful fraction in reducing free radical activity carried by DPPH compared to the methanol fraction with an IC_50_ value of 22.59 ± 3.41 mg/ml. The three Temulawak fractions are known to have good antibacterial activity, yet the ethyl acetate fraction has a better ability to kill the test bacteria compared to the methanol and n-hexane fractions. The minimum concentration required by the ethyl acetate fraction is 6.25 mg/ml for all types of bacteria.

## List of abbreviations

DPPH, (2,2-diphenyl-1-picrylhydrazyl); EtOAc, ethyl acetate; IC_50_, inhibitory concentration; MBC, minimum bactericidal concentration; MeOH, methanol; mg, milligram; mg GA/extract, milligram gallic acid equivalent; MIC, minimum inhibitory concentration; ml, milliliter; mm, millimeter; nm, nanometer; μl, microliter; PPM, parts per million; R^2^, correlation coefficient value; SGP, step gradient polarity.
